# G6PD facilitates axon regeneration *via* clathrin-mediated endocytosis

**DOI:** 10.1016/j.jbc.2026.111345

**Published:** 2026-03-04

**Authors:** Chunyi Jiang, Xinyi Liu, Hui Li, Yan Lu, Qianqian Cao, Bin Yu, Susu Mao

**Affiliations:** Jiangsu Key Laboratory of Tissue Engineering and Neuroregeneration, Key Laboratory of Neuroregeneration of Ministry of Education, Affiliated Hospital of Nantong University, Co-Innovation Center of Neuroregeneration, Nantong University, Nantong, China

**Keywords:** axon regeneration, G6PD, endocytosis, clathrin, peripheral nerve injury, metabolic reprogramming

## Abstract

Metabolic reprogramming is a hallmark of neuronal repair, yet the roles of glucose metabolism-related enzymes remain poorly understood. To investigate their functions, we employed a sciatic nerve injury model, taking advantage the intrinsic regenerative capacity of peripheral neurons. After sciatic nerve crush injury, dorsal root ganglia exhibited sustained upregulation of several enzymes in the pentose phosphate pathway. Notably, glucose-6-phosphate dehydrogenase (G6PD), the rate-limiting enzyme of the pentose phosphate pathway, was markedly increased at both RNA and protein levels. Silencing G6PD impaired axon regeneration *in vitro* and *in vivo*, whereas its overexpression enhanced regrowth. Interestingly, G6PD overexpression did not alter the NADP^+^/NADPH ratio, suggesting a nonmetabolic role. Using mass spectrometry, coimmunoprecipitation, and Duolink proximity ligation assays, we identified clathrin heavy chain as a specific binding partner of G6PD. Mechanistic analyses further showed that G6PD facilitated neuronal endocytosis through direct interaction with clathrin heavy chain, thereby promoting axon regeneration. These findings identify G6PD as a molecular link between metabolic reprogramming and membrane trafficking, revealing an unexpected nonmetabolic role in neural repair.

Metabolism comprises a precisely coordinated network of chemical reactions that are fundamental to life. Beyond providing essential substrates and energy, it also serves as a key regulator of gene expression. Through metabolic enzymes and their products, metabolites, metabolism exerts profound influence over cell growth, survival, differentiation, and the maintenance of systemic homeostasis (1, 2). Metabolic reprogramming, the dynamic alteration of cellular metabolic pathways, enables cells to adapt to changing energy demands and biosynthetic needs under physiological or pathological conditions (3).

In recent years, increasing attention has been focused on the role of metabolic reprogramming in neural repair (4, 5, 6). Neural regeneration requires not only substantial energy but also an abundant supply of lipids, as well as the synthesis of proteins and nucleic acids, all of which depend on reprogrammed metabolic processes after injury and are driven by specific metabolic enzymes (7, 8, 9, 10). Several of these enzymes have been identified as critical regulators of axonal regeneration. For instance, PTEN, a lipid phosphatase, dephosphorylates phosphatidylinositol (3,4,5)-trisphosphate (PIP3) into phosphatidylinositol (4,5)-bisphosphate, thereby negatively regulating the PI3K pathway. The PTEN–PI3K signaling axis has been well established as a key modulator of axonal regeneration, as deletion of PTEN significantly enhances regenerative capacity within the central nervous system (CNS) (11, 12). Similarly, Anja *et al*. identified plasticity-related gene 1, which encodes a membrane-associated lipid phosphate phosphatase expressed in neurons and localized to growing axonal membranes. Plasticity-related gene 1 acts as an ectoenzyme, counteracting phospholipid-induced axonal collapse and promoting hippocampal axon elongation (13). In addition, deletion of LPIN1, a phosphatidic acid phosphatase, enhances optic and sciatic nerve repair by lowering triglycerides, promoting phospholipid synthesis, and remodeling lipid metabolism (5). We further showed that LPIN2, another family member, supports regeneration in dorsal root ganglia (DRG) neurons by increasing triglyceride-enriched lipid droplets (14). Moreover, our previous work identified pyruvate dehydrogenase beta subunit, a core tricarboxylic acid (TCA) cycle enzyme, as a facilitator of axonal regeneration through coordinated regulation of energy metabolism and epigenetic reprogramming (15). Collectively, these findings highlight that targeting metabolic enzymes to induce metabolic reprogramming represents a promising therapeutic strategy for promoting axonal regeneration after neural injury.

Glucose metabolism is a fundamental pathway that supports cellular energy production and biosynthesis. Upon uptake through glucose transporters, glucose is catabolized *via* three major pathways, including glycolysis, the pentose phosphate pathway (PPP), and the TCA cycle. Although glucose metabolism is essential for cellular maintenance and repair, its specific role in axonal regeneration remain largely undefined. Recent studies highlighted injury-induced upregulation of glycolysis as a mechanism promoting axon regeneration (4), yet the contribution of individual metabolic enzymes requires further elucidation.

In contrast to CNS neurons, peripheral neurons retain a strong intrinsic ability to regenerate their axons following injury, making them a valuable model for uncovering underlying molecular mechanisms of axonal regeneration. In the present study, we analyzed RNA-seq datasets from rat DRG after sciatic nerve crush injury (16) and identified marked upregulation of enzymes involved in the PPP. Among these, we focused on glucose-6-phosphate dehydrogenase (G6PD), the rate-limiting enzyme of the PPP, and further investigated its role in axonal regeneration in DRG neurons. Surprisingly, despite its classical metabolic function, our findings suggest that G6PD promotes axonal regeneration through a nonmetabolic mechanism.

## Results

### G6PD is required for axonal regeneration in DRG neurons

**Figure 1:**
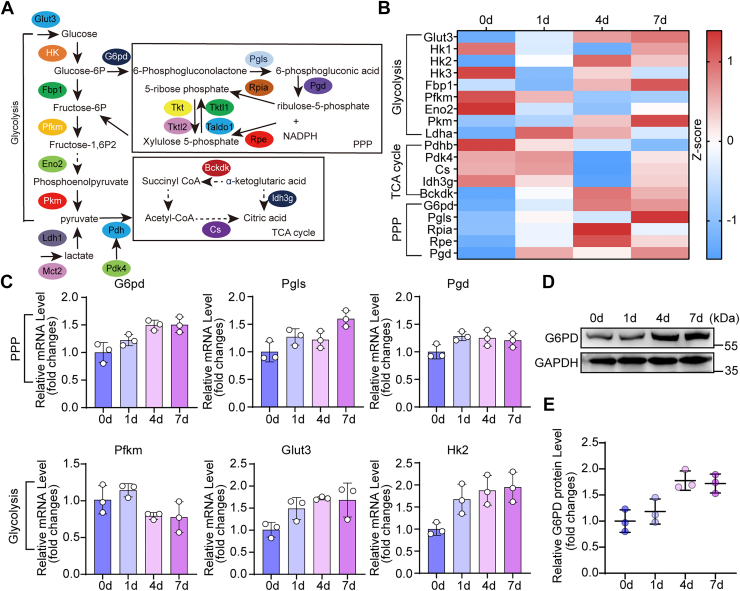
**Expression analysis of glucose metabolic enzymes in dorsal root ganglia following sciatic nerve crush injury in rats**. *A*, schematic diagram illustrating glucose metabolic pathways, including glycolysis, the TCA cycle, and pentose phosphate pathway (PPP). *B*, heat map displaying mRNA expression patterns of the key glucose metabolic enzymes in dorsal root ganglia at various time points post-sciatic nerve injury (SNI). *C*, qRT-PCR analysis of mRNA expression changes of key enzymes involved in the PPP and Glycolysis at different time points after SNI (mean ± SD; n = 3 biologically independent experiments). *D*, Western blotting analysis of temporal changes in G6PD expression after SNI. *E*, quantification of G6PD protein levels relating to (D) (mean ± S.D.; n = 3 biologically independent experiments). G6PD, glucose-6-phosphate dehydrogenase; TCA, tricarboxylic acid.

To identify key glucose metabolic enzymes involved in axon regeneration (Fig. 1*A*), we first analyzed RNA-seq data from rat DRG following sciatic nerve injury (SNI) (16). This analysis revealed that gene expression in glycolysis exhibits dynamic fluctuations, whereas expression of genes in the TCA cycle progressively declines over the time course of SNI. In contrast, genes involved in the PPP show a sustained increase, particularly during a critical window for axon elongation (Fig. 1*B*) (17). We next selected genes with variable expression after SNI and validated the sequencing results by quantitative real-time PCR (Fig. 1*C*). Given that G6PD is the rate-limiting enzyme of the PPP, we performed Western blotting, which confirmed a sustained increase in G6PD protein levels (Fig. 1, *D* and *E*). Based on this evidence, we selected G6PD for further investigation.

**Figure 2:**
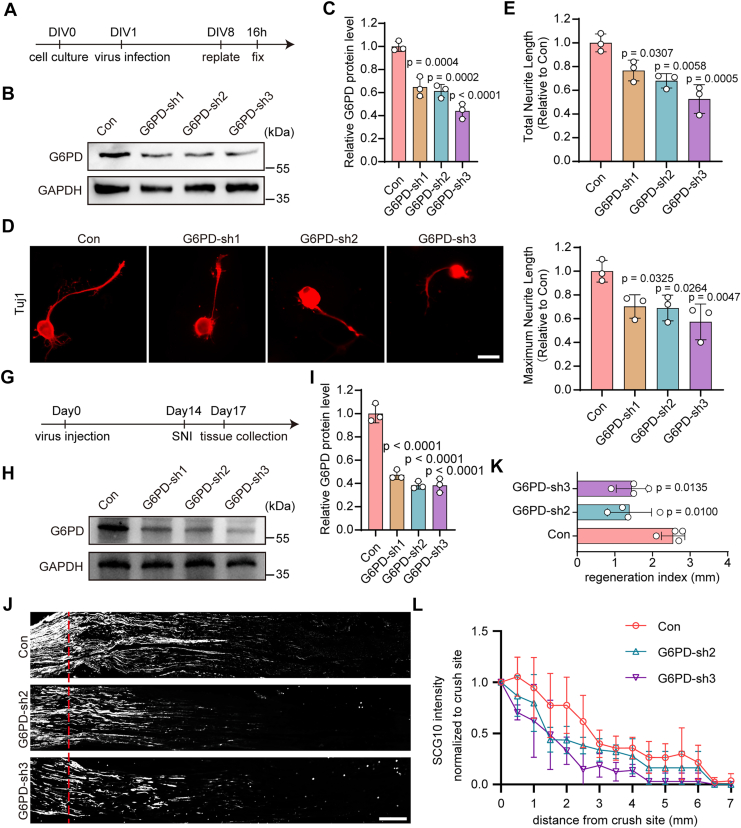
**G6PD knockdown impairs axonal regeneration in DRG neurons**. *A*, timeline for AAV-mediated G6PD knockdown and replating-induced axotomy in primary DRG neurons. *B*, Western blotting analysis of G6PD expression in DRG neurons infected with control AAV2/8-expressing scramble shRNA (Con) or AAV-expressing shRNA1 (G6PD-sh1), G6PD-sh2, or G6PD-sh3 to knock down G6PD. *C*, quantification of G6PD protein levels relating to (*B*) (mean ± SD; one-way ANOVA, Dunnett *post hoc* test, n = 3 biologically independent experiments). *D*, representative images of replated primary DRG neurons after G6PD knockdown, labeled with Tuj-1. The scale bar represents 20 μm. *E* and *F*, quantitative analysis of total and maximum neurite length per neuron relating to *panel* (*D*), normalized to Con group (mean ± SD; one-way ANOVA, Dunnett *post hoc* test, n = 3 biologically independent experiments). *G*, timeline for G6PD knockdown *in vivo*, SNI and tissue collection. *H*, Western blotting analysis of G6PD expression in DRG tissues infected with Con or G6PD-sh1, G6PD-sh2, and G6PD-sh3 AAV. *I*, quantification of G6PD protein levels relating to (*H*) (mean ± SD; one-way ANOVA, Dunnett *post hoc* test, n = 3 biologically independent experiments). *J*, representative longitudinal sections from injured sciatic nerves. The crush site is indicated by a *red dotted line*. The scale bar represents 500 μm. *K*, axon regeneration in injured rats was quantified by regeneration indices obtained from SCG10 immunostaining on day 3 after injury (mean ± SD; one-way ANOVA, Dunnett *post hoc* test, n = 4 rats per group). *L*, normalized SCG10 intensity plotted in function of the distance from the crush line (n = 4 rats per group). DRG, dorsal root ganglia; G6PD, glucose-6-phosphate dehydrogenase.

To determine whether G6PD contributes to axonal regeneration, we first inhibited its activity using 6-aminonicotinamide (6AN) in an *in vitro* neurite regrowth assay that models regeneration after peripheral axotomy (18, 19). Tuj1 immunostaining showed that 10 μM, but not 1 μM, 6AN significantly reduced both total and maximum neurite length compared to the DMSO control (Fig. S1, *A*–*C*). To further validate the role of G6PD, we used AAV2/8-mediated delivery of G6PD-specific shRNAs to knock down its expression in cultured DRG neurons. DRG cultures were infected at day 1 *in vitro* (Fig. 2*A*), and Western blotting performed 7 days later confirmed effective knockdown of G6PD by three independent shRNAs (G6PD-sh1, G6PD-sh2, and G6PD-sh3) (Fig. 2, *B* and *C*). Neurite regrowth assay on replated DRG neurons revealed that G6PD knockdown significantly impaired both total and maximum neurite extension across all shRNA conditions compared with control shRNA (Fig. 2, *D*–*F*).

To assess the *in vivo* role of G6PD in axonal regeneration, we delivered AAV2/8 carrying G6PD-shRNA intrathecally into rats subjected to SNI (Fig. 2*G*). This approach effectively reduced G6PD expression in DRGs (Fig. 2, *H* and *I*). We then chose two shRNAs exhibiting greater knockdown efficiency (G6PD-sh2 and G6PD-sh3) for subsequent *in vivo* functional validation. Three days postinjury, axonal regeneration was evaluated using SCG10, a well-established marker of regenerating sensory axons (20). G6PD knockdown markedly diminished SCG10^+^ axonal extension compared to control shRNA-treated animals (Fig. 2*J*). Quantification using the regeneration index, defined as the distance from the crush site where SCG10 intensity declined to 50% of that at the injury site (21), confirmed a significant reduction in knockdown animals (Fig. 2, *K* and *L*). Together, these findings demonstrate that G6PD is essential for axonal regeneration in DRG neurons both *in vitro* and *in vivo*.

### Overexpression of G6PD enhances axonal regeneration in DRG neurons

**Figure 3:**
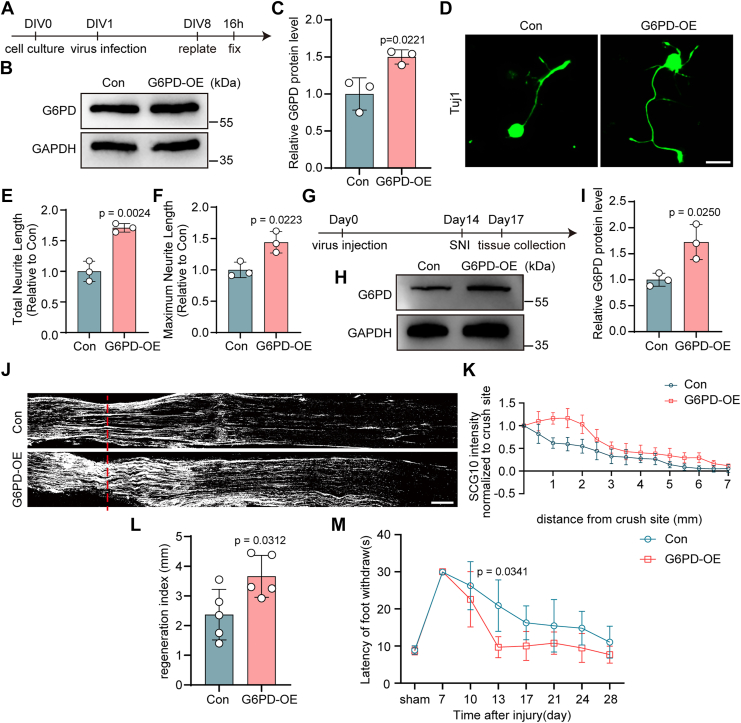
**G6PD overexpression enhances nerve regeneration and sensory recovery**. *A*, timeline for AAV-mediated G6PD overexpression and replating-induced axotomy in primary DRG neurons. *B*, Western blotting analysis of G6PD expression in DRG neurons infected with control AAV2/8 (Con), or AAV-overexpressing rat G6PD (G6PD-OE). *C*, quantification of G6PD protein levels relating to (*B*) (mean ± SD; unpaired two-tailed *t* test, n = 3 biologically independent experiments). *D*, representative images of replated primary DRG neurons after G6PD overexpression, labeled with Tuj-1. The scale bar represents 20 μm. *E* and *F*, quantitative analysis of total and maximum neurite length per neuron relating to panel (*D*), normalized to Con group (mean ± SD; unpaired two-tailed *t* test, n = 3 biologically independent experiments). *G*, timeline for G6PD overexpression *in vivo*, SNI and tissue collection. *H*, Western blotting analysis of G6PD expression in DRG tissues infected with Con or G6PD-OE AAV. *I*, quantification of G6PD protein levels relating to (*H*) (mean ± SD; unpaired two-tailed *t* test, n = 3 biologically independent experiments). *J*, representative longitudinal sections from injured sciatic nerves. The crush site is indicated by a *red dotted line*. The scale bar represents 500 μm. *K*, normalized SCG10 intensity plotted in function of the distance from the crush line (n = 5 rats per group). *L*, axon regeneration in injured rats was quantified by regeneration indices obtained from SCG10 immunostaining on day 3 after injury (mean ± SD; unpaired two-tailed *t* test, n = 5 rats per group). *M*, assessment of the recovery of thermal sensory function after SNI in rats infected with Con or G6PD-OE AAV (mean ± SD; unpaired two-tailed *t* test, n = 7–8 rats per group). DRG, dorsal root ganglia; G6PD, glucose-6-phosphate dehydrogenase; SNI, sciatic nerve injury.

To assess whether upregulating G6PD enhances axonal regeneration in DRG neurons, we overexpressed rat G6PD using an AAV2/8 vector driven by the human synapsin promoter. Western blotting analysis confirmed successful G6PD overexpression in primary DRG neurons 7 days postinfection (Fig. 3, *A*–*C*). Neurite regrowth assay revealed that G6PD overexpression significantly increased both the total and maximum neurite length of replated DRG neurons compared with control virus-treated cells (Fig. 3, *D*–*F*).

To evaluate the *in vivo* effect of G6PD overexpression, AAV2/8-G6PD was delivered intrathecally into rats (Fig. 3*G*). Western blotting confirmed increased G6PD protein levels in DRG tissue following viral delivery (Fig. 3, *H* and *I*). Anatomically, SCG10-positive axonal extension was markedly enhanced in the G6PD overexpression group relative to controls after SNI (Fig. 3, *J* and *K*), with a significantly higher regeneration index than controls, indicating improved axonal regeneration (Fig. 3*L*).

Following sciatic nerve crush injury, regenerating peripheral axons extend toward the epidermis and initiate cutaneous reinnervation in the hind paw 2 to 3 weeks after crush injury (22). To assess functional recovery, we compared sensory responses in rats with or without G6PD overexpression. Thermal sensitivity, measured by heat-evoked withdrawal latency, exhibited accelerated recovery in G6PD-overexpressing animals, with improvements evident by day 10 and reaching significance by day 13 (Fig. 3*M*). Together, these findings suggest that G6PD overexpression enhances both structural axon regeneration and functional recovery after peripheral nerve injury.

### G6PD directly binds to CLTC in DRG neurons

**Figure 4:**
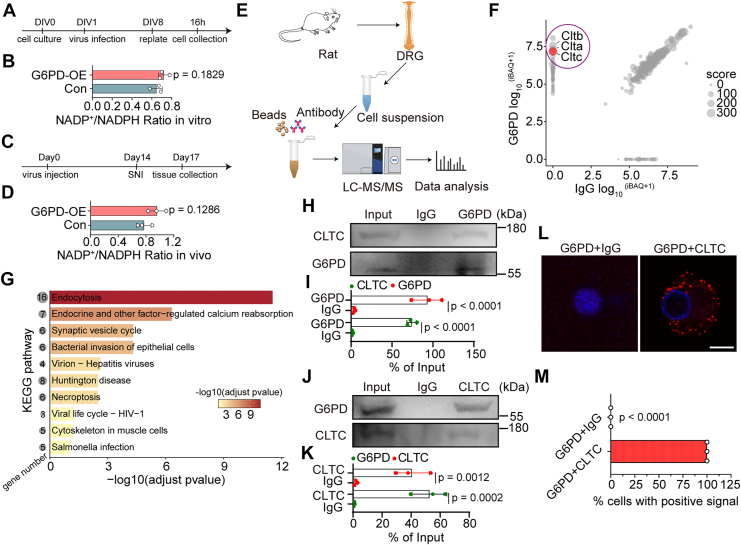
**G6PD directly binds to CLTC**. *A*, timeline for AAV-mediated G6PD overexpression and replating-induced axotomy in primary DRG neurons. *B*, NADP^+^/NADPH ratio following G6PD overexpression in resuspended DRG neurons (mean ± SD; unpaired two-tailed *t* test, n = 3 biologically independent experiments). *C*, timeline for G6PD overexpression *in vivo*, SNI and tissue collection. *D*, NADP^+^/NADPH ratio following G6PD overexpression in DRG tissues 3 days post-SNI (mean ± SD; unpaired two-tailed *t* test, n = 3 rats per group). *E*, identification of G6PD-binding proteins by coimmunoprecipitation (Co-IP) and mass spectrometry. *F*, spectral counts of G6PD-interacting proteins identified by mass spectrometry after Co-IP from DRG tissue extracts. *Purple ellipse* indicates G6PD-specific interactors (iBAQ > 0 in G6PD group and iBAQ = 0 in IgG group). *G*, enriched pathways of G6PD-specific interactors. The size of the *circles* on the *left* represents the number of enriched genes in each pathway. *H* and *J*, Co-IP and Western blotting validation of G6PD–CLTC interaction. *I* and *K*, quantification of protein levels immunoprecipitated with the indicated antibodies for (*H*) and (*J*), shown as % of input. The *p* values represent comparisons between target antibody and IgG control (mean ± SD; two-way ANOVA, Bonferroni *post hoc* test, n = 3 biologically independent experiments). *L*, Duolink assay confirming G6PD–CLTC direct interaction in DRG neurons. The scale bar represents 10 μm. *M*, quantification of Duolink PLA signals. G6PD antibody paired with IgG (control) or CLTC. No PLA foci in G6PD+IgG group (0/60 cells); all G6PD+CLTC cells positive (60/60 cells). Data are % PLA-positive cells from three independent experiments (20 cells/experiment, Fisher's exact test). CLTC, clathrin heavy chain; DRG, dorsal root ganglia; G6PD, glucose-6-phosphate dehydrogenase; PLA, proximity ligation assay; SNI, sciatic nerve injury.

The PPP is a well-established antioxidant pathway that generates NADPH, which maintains glutathione level and counteracts reactive oxygen species (ROS) (23). ROS levels in DRG neurons rise within 2 h after SNI, remain elevated for approximately 24 h, and return to baseline levels at later stages (3–7 days) (24). Notably, G6PD expression was predominantly upregulated at 4 and 7 days after SNI, suggesting a potential role beyond NADPH production. To test this, we first examined its canonical metabolic function, NADPH generation *via* the PPP. G6PD was overexpressed in primary DRG neurons using AAV, and NADP^+^/NADPH ratio was measured after simulated axonal injury *in vitro* (Fig. 4*A*). The results showed no significant difference in NADP^+^/NADPH ratio between G6PD-overexpressing and control groups (Fig. 4*B*). Similarly, *in vivo* analysis 3 days post-SNI revealed no detectable increase in NADP^+^/NADPH ratio in DRG tissue of G6PD-overexpressing animals (Fig. 4, *C* and *D*). These findings suggest that G6PD enhances axon regeneration *via* a mechanism independent of its classic metabolic function in the PPP.

Previous studies suggest that G6PD may exert noncanonical effects through direct protein–protein interactions (25). To explore this, we isolated DRG tissues and performed coimmunoprecipitation (Co-IP) followed by mass spectrometry (MS) using a G6PD-specific antibody to identify its binding partners (Fig. 4*E*, Table S1). This analysis identified 63 proteins with strong binding specificity (Fig. 4*F*), among which functional enrichment analysis revealed endocytosis as the most significantly represented pathway, with 16 associated proteins (Fig. 4*G*, Table S2). Notably, the clathrin subunits CLTA, CLTB, and particularly CLTC, the clathrin heavy chain with the highest score, were enriched. CLTC encodes the core scaffold of clathrin-mediated endocytosis (CME) and vesicle trafficking (26). Subsequent Co-IP assays confirmed the interaction between G6PD and CLTC (Fig. 4, *H*–*K*). To further validate and localize this interaction *in situ*, we employed Duolink proximity ligation assay (PLA), a highly sensitive method capable of detecting endogenous protein–protein interactions within 40 nm (27). PLA revealed abundant G6PD–CLTC interactions, primarily localized to the cytoplasm of DRG neurons (Fig. 4, *L* and *M*). Together, these findings demonstrate that G6PD directly binds CLTC, implicating a nonmetabolic, protein interaction–based mechanism in its proregenerative function.

### Suppression of CLTC-mediated endocytosis inhibits axonal regeneration in neurons

**Figure 5:**
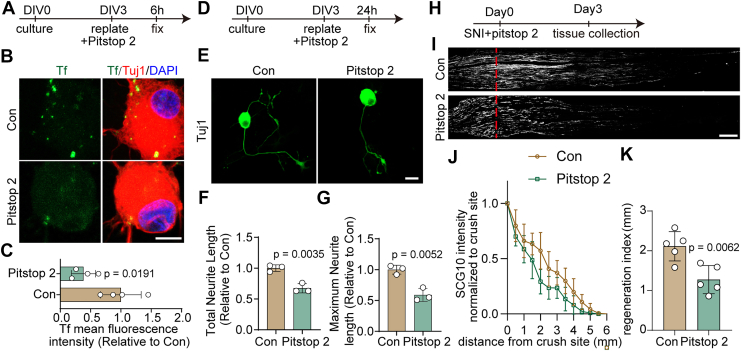
**Inhibiting endocytosis by Pitstop 2 impairs axonal regeneration**. *A*, timeline for Pitstop 2 treatment upon replating-induced axotomy in primary DRG neurons. *B*, DRG neurons were incubated with Tf-Alexa 488 (*green*) and immunostained for Tuj1 (*red*) as neuronal marker and DAPI (*blue*). The scale bar represents 10 μm. *C*, quantification of mean fluorescence intensity of endocytosed transferrin for the experiment shown in (*B*), normalized to the control (Con) group (mean ± SD; unpaired two-tailed *t* test, n = 4 biologically independent experiments). *D*, timeline for Pitstop 2 treatment upon replating-induced axotomy in primary DRG neurons. *E*, representative images of replated primary DRG neurons after Pitstop 2 treatment, labeled with Tuj-1 (*green*). DMSO treatment served as the control (Con). The scale bar represents 20 μm. *F* and *G*, quantitative analysis of total and maximum neurite length per neuron relating to panel (*E*), normalized to Con group (mean ± SD; unpaired two-tailed *t* test, n = 3 biologically independent experiments). *H*, timeline for Pitstop 2 treatment *in vivo*, SNI and tissue collection. *I*, representative longitudinal sections from injured sciatic nerves. The crush site is indicated by a *red dotted line*. The scale bar represents 500 μm. *J*, normalized SCG10 intensity plotted in function of the distance from the crush line (n = 5 rats per group). *K*, axon regeneration in injured rats was quantified by regeneration indices obtained from SCG10 immunostaining on day 3 after injury (mean ± SD; unpaired two-tailed *t* test, n = 5 rats per group). CLTC, clathrin heavy chain; DRG, dorsal root ganglia; G6PD, glucose-6-phosphate dehydrogenase; SNI, sciatic nerve injury; Tf, transferrin.

Endocytosis is critical for neurotrophin-driven dendrite branching (28) and Schwann cell–mediated axonal regeneration (29). However, the specific role of neuronal CME in axon regeneration remains poorly defined. To investigate this, we used Pitstop 2, a selective inhibitor of CME (30). Transferrin receptor–mediated endocytosis is the best-characterized assay for CME, and DRG neurons have been reported to internalize transferrin *via* CME (31). We therefore first validated the inhibitory efficiency of Pitstop 2 on this pathway (Fig. 5*A*). Transferrin uptake assay confirmed that Pitstop 2 treatment significantly inhibited CME in primary DRG neurons (Fig. 5, *B* and *C*). Having established its inhibitory effect, we then applied Pitstop 2 in an *in vitro* axonal injury model. Primary DRG neurons were cultured for 3 days and treated with Pitstop 2 in an *in vitro* axonal injury model (Fig. 5*D*). Tuj1 staining revealed a significant reduction in both total and maximum neurite length following Pitstop 2 treatment, indicating impaired axonal regrowth (Fig. 5, *E*–*G*).

To evaluate the *in vivo* effects of Pitstop 2, intrathecal administration was performed at the time of SNI (Fig. 5*H*). SCG10 immunostaining revealed a marked decrease in regenerating axons in Pitstop 2–treated animals compared to controls (Fig. 5, *I* and *J*). Quantitative analysis demonstrated a significantly reduced regeneration index (Fig. 5*K*), supporting the conclusion that inhibition of CME impairs axonal regeneration *in vivo*.

**Figure 6:**
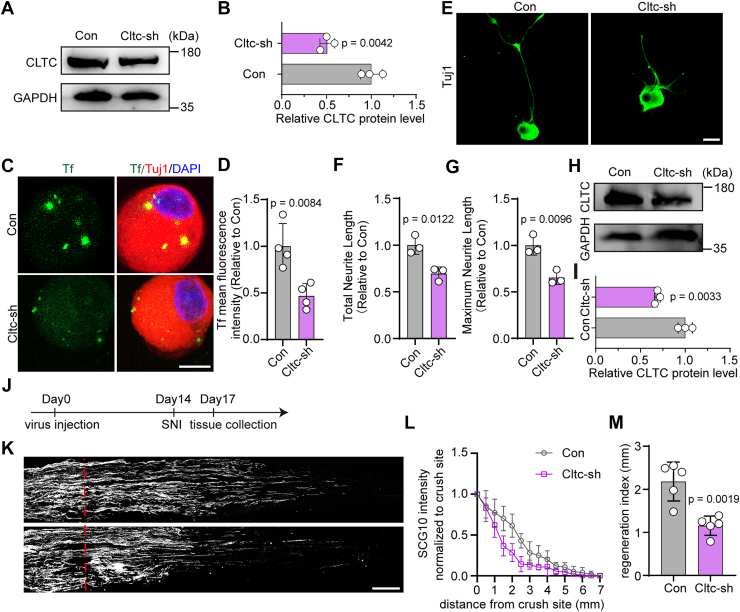
**CLTC knockdown impairs axonal regeneration in DRG neurons**. *A*, Western blotting analysis of CLTC protein expression in DRG neurons infected with control AAV2/8-expressing scramble shRNA (Con) or AAV-expressing shRNA (Cltc-sh) to knock down CLTC. *B*, quantification of CLTC protein levels relating to (*A*) (mean ± SD; unpaired two-tailed *t* test, n = 3 biologically independent experiments). *C*, DRG neurons were infected with AAV2/8 as in (*A*). After 7 days of infection, neurons were replated, cultured for 6 h, then incubated with Tf-Alexa 488 (*green*), and immunostained for Tuj1 (*red*) and DAPI (*blue*). The scale bar represents 10 μm. *D*, quantification of mean fluorescence intensity of endocytosed transferrin for the experiment shown in (*C*), normalized to the control (Con) group (mean ± SD; unpaired two-tailed *t* test, n = 4 biologically independent experiments). *E*, representative images of replated primary DRG neurons after CLTC knockdown, labeled with Tuj-1 (*green*). The scale bar represents 20 μm. *F* and *G*, quantitative analysis of total and maximum neurite length per neuron in *panel* (*E*), normalized to Con group (mean ± SD; unpaired two-tailed *t* test, n = 3 biologically independent experiments). *H*, Western blotting analysis of CLTC expression in DRG tissues infected with Con or Cltc-sh AAV. *I*, quantification of CLTC protein levels relating to (*H*) (mean ± SD; unpaired two-tailed *t* test, n = 3 biologically independent experiments). *J*, timeline for virus injection *in vivo*, SNI and tissue collection. *K*, representative longitudinal sections from injured sciatic nerves. The crush site is indicated by a *red dotted line*. The scale bar represents 500 μm. *L*, normalized SCG10 intensity plotted in function of the distance from the crush line (n = 5 rats per group). *M*, axon regeneration in injured rats was quantified by regeneration indices obtained from SCG10 immunostaining on day 3 after injury (mean ± SD; unpaired two-tailed *t* test, n = 5 rats per group). CLTC, clathrin heavy chain; DRG, dorsal root ganglia; G6PD, glucose-6-phosphate dehydrogenase; SNI, sciatic nerve injury.

To genetically validate the role of CLTC, we constructed AAV2/8 vectors carrying CLTC-targeting shRNAs (32) and infected primary DRG neurons *in vitro*. Western blotting confirmed efficient knockdown of CLTC protein expression (Fig. 6, *A* and *B*). Transferrin uptake assay further confirmed that *Cltc* silencing significantly suppressed CME activity in DRG neurons (Fig. 6, *C* and *D*). In replated neurons, Tuj1 immunostaining showed that *Cltc* silencing significantly reduced axonal growth in cultured DRG neurons (Fig. 6, *E*–*G*). *In vivo*, intrathecal delivery of AAV2/8-CLTC-shRNA effectively reduced CLTC protein levels in DRGs within 2 weeks (Fig. 6, *H* and *I*). Following SNI, axon regeneration was assessed 3 days later (Fig. 6*J*). SCG10 staining showed shortened regenerating axons with a reduced regeneration index (Fig. 6, *K*–*M*). These findings collectively establish CLTC-mediated endocytosis as critical for axonal regeneration in DRG neurons.

### G6PD promotes axonal regeneration through CLTC-mediated endocytosis

**Figure 7:**
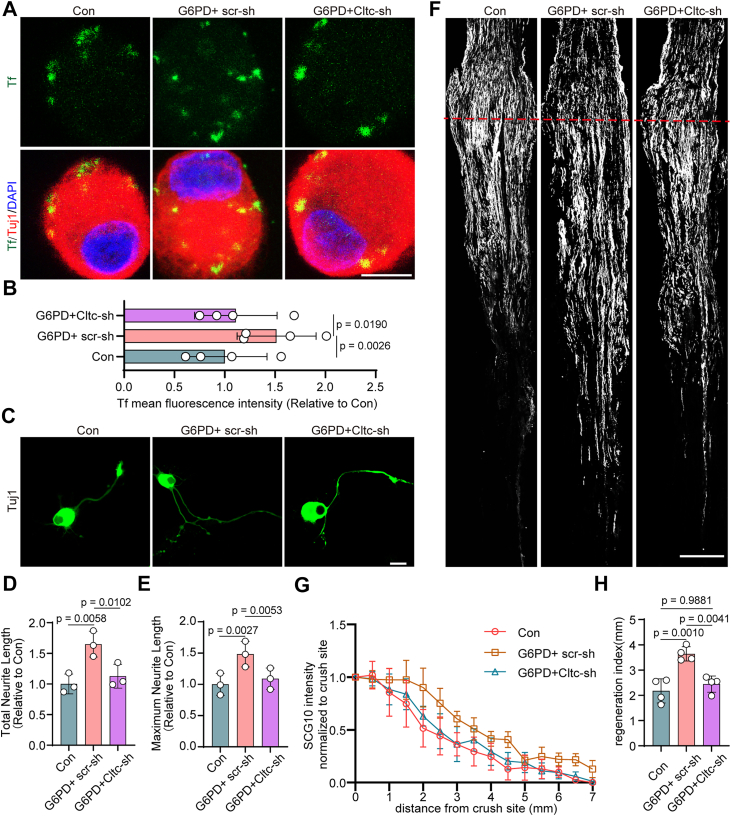
**G6PD enhances axonal regeneration through CLTC-mediated endocytosis**. *A*, primary DRG neurons were infected with AAVs as follows: control AAV-expressing scramble shRNA alone (Con); AAV-overexpressing G6PD together with AAV-expressing scramble shRNA (G6PD+scr-sh); or AAV-overexpressing G6PD together with AAV-expressing shRNA targeting Cltc (G6PD+Cltc-sh). After 7 days of infection, neurons were replated, cultured for another 6 h, and then incubated with Tf-Alexa 488 (*green*) and immunostained for the neuronal marker Tuj1 (*red*) and DAPI (*blue*). The scale bar represents 10 μm. *B*, quantification of mean fluorescence intensity of endocytosed transferrin for the experiment shown in (*A*), normalized to the control (Con) group (mean ± SD; one-way ANOVA, Bonferroni *post hoc* test, n = 4 biologically independent experiments). *C*, representative images of replated primary DRG neurons infected with AAV2/8 as in (*A*). Neurons were infected for 7 days, replated, cultured for another 16 h, then fixed and labeled with Tuj-1 (*green*). The scale bar, 20 μm. *D* and *E*, quantitative analysis of total and maximum neurite length per neuron relating to *panel* (*C*), normalized to Con group (mean ± SD; one-way ANOVA, Bonferroni *post hoc* test, n = 3 biologically independent experiments). *F*, representative longitudinal sections from injured sciatic nerves. The crush site is indicated by a *red dotted line*. The scale bar represents 500 μm. *G*, normalized SCG10 intensity plotted in function of the distance from the crush line (n = 4 rats per group). *H*, axon regeneration in injured rats was quantified by regeneration indices obtained from SCG10 immunostaining on day 3 after injury (mean ± SD; one-way ANOVA, Bonferroni *post hoc* test, n = 4 rats per group). Note that the G6PD-induced increase in the regeneration index was not maintained upon concomitant CLTC knockdown, with values returning to levels comparable to the control group. CLTC, clathrin heavy chain; DRG, dorsal root ganglia; G6PD, glucose-6-phosphate dehydrogenase.

Given that G6PD directly binds CLTC, we hypothesized that its proregenerative effects are dependent on CME. To test this, we transfected DRG neurons with AAV2/8-expressing scramble shRNA (Con), AAV2/8-G6PD, and AAV2/8-expressing scramble shRNA (G6PD+scr-sh), or AAV2/8-G6PD and AAV2/8-CLTC-shRNA (G6PD+Cltc-sh), respectively. To directly assess whether G6PD enhances CME in a CLTC-dependent manner, we performed transferrin uptake assays in DRG neurons under the indicated experimental conditions. G6PD overexpression significantly increased transferrin internalization compared with control neurons, indicative of enhanced CME activity. This increase was largely abolished by co-knockdown of CLTC (Fig. 7, *A* and *B*), confirming the dependence of G6PD's proendocytic effect on CLTC. In the *in vitro* axon regrowth assay, G6PD overexpression enhanced neurite outgrowth and endocytosis as expected. However, co-knockdown of CLTC significantly abolished this enhancement, demonstrating that G6PD's effect on axon regeneration requires CLTC (Fig. 7, *C*–*E*). To further validate this *in vivo*, we coadministered AAV2/8-G6PD and AAV2/8-CLTC-shRNA intrathecally in rats. Following SNI, G6PD overexpression alone enhanced axonal regeneration (Fig. 7, *F*–*H*). In contrast, concomitant CLTC knockdown largely abolished these beneficial effects, such that these parameters were no longer significantly different from control levels (Fig. 7*H*).

Together, these results demonstrate that G6PD facilitates axonal regeneration through a CLTC-mediated endocytosis mechanism, highlighting a previously unrecognized nonmetabolic function of G6PD in neuronal repair.

## Discussion

In this study, we characterized glucose metabolic enzyme expression after SNI in rats and identified sustained upregulation of G6PD, the key enzyme of the PPP, from day 1 to day 7 postinjury. Functional assays *in vitro* and *in vivo* established G6PD as a critical regulator of axonal regeneration in DRG neurons. Mechanistically, G6PD interacts directly with CLTC, exerting a PPP-independent, nonmetabolic role in controlling neuronal endocytosis. Our study therefore reveals a novel role of a metabolic enzyme, G6PD, in modulating intracellular endocytosis to drive axonal regeneration.

Although glucose metabolism is crucial for neuronal function, its role in axonal regeneration remains poorly understood. Recently, Liu *et al*. found that harmine, a small molecule enhancing glucose metabolism and mitochondrial energy supply, promotes axon regeneration by addressing the energy deficit postinjury (33). In addition to providing the necessary substrates and energy for axon regeneration after injury, we speculate that glucose metabolism, particularly the PPP, plays a crucial role in maintaining redox homeostasis through NADPH production, thereby protecting neurons from oxidative stress. This notion is supported by our observation that multiple PPP-related enzymes in DRG were markedly upregulated after SNI. As the rate-limiting enzyme of the PPP, G6PD is well known to safeguard red blood cells against oxidative stress, and its deficiency causes hereditary hemolytic anemia (34). Moreover, G6PD is highly expressed in many tumor types, where it contributes to antioxidant defense (35). Consistent with these roles, our data demonstrate that G6PD is indispensable for sciatic nerve regeneration.

Interestingly, however, G6PD overexpression did not further reduce the postinjury NADP^+^/NADPH ratio, yet axon regeneration was still enhanced. Furthermore, we also observed that G6PD expression remained elevated from day 1 to day 7 postinjury. However, previous studies have shown that while ROS levels in DRG neurons rise rapidly after SNI, they largely return to baseline by day 3 (24). Taken together, these findings suggest a stage-dependent function of G6PD following SNI: in the early stage, it primarily acts to maintain redox homeostasis, whereas in the later stage, its nonmetabolic function in facilitating CME becomes critical for axon regeneration. This temporal transition highlights a biphasic model, in which, G6PD sequentially fulfills distinct functions, first as a metabolic guardian and then as a structural promoter of membrane dynamics, to promote injury repair. An important question for future studies is to identify the molecular switch that mediates this functional transition from a metabolic enzyme to a nonmetabolic scaffold.

Gene enrichment analysis in the present work revealed that numerous proteins potentially interacting specifically with G6PD are involved in endocytosis. Further mechanistic investigation collectively demonstrates that G6PD promotes axonal regeneration in a PPP-independent manner by directly modulating CLTC-mediated endocytosis. This identified G6PD–CLTC–endocytosis axis raises intriguing questions regarding how clathrin-dependent vesicular trafficking influences axonal growth. Endocytosis is a fundamental cellular process through which cells internalize extracellular molecules, plasma membrane components, and surface receptors by engulfing them within membrane-bound vesicles. This mechanism critically regulates nutrient uptake, signal transduction, and membrane homeostasis. In neurons, CME represents a major endocytic mechanism (36). During CME, clathrin and adapter proteins assemble into a coat around a membrane invagination, forming a clathrin-coated pit. These pits subsequently undergo dynamin-mediated scission, releasing clathrin-coated vesicles that deliver cargo to early endosomes for sorting and processing (26). Previous research demonstrates that endocytosis controls dendrite branching, maintains neuronal polarity, and mediates plasticity at the axon initial segment (28, 37). However, its role in axon regeneration postinjury is undetermined. A recent investigation detected altered expression in multiple endocytosis-associated genes following SNI, implying their potential functional involvement in peripheral nerve repair mechanisms (29). Furthermore, *in vitro* studies have established the involvement of endocytic mechanisms in axonal growth cone remodeling and receptor recycling (38). Building upon these findings, we propose that G6PD may act as a scaffold protein facilitating the assembly of clathrin-coated pits, thereby regulating the internalization of inhibitory signals (*e*.*g*., Nogo receptors) or the recycling of proregenerative receptors (*e*.*g*., TrkB). This hypothesis is supported by our observation that inhibition of CLTC-mediated endocytosis abolished G6PD's regenerative effects. These results suggest that G6PD promotes axonal repair through both metabolic support, which helps meet bioenergetic demands, and regulation of membrane dynamics, which facilitates the removal of inhibitory signals and the recycling of growth-promoting receptors. However, the specific effector molecules through which G6PD/CLTC mediates axonal regeneration remain to be elucidated and warrant in-depth investigation.

Also, clathrin participates in post-Golgi trafficking and lysosomal enzyme delivery, which were not assessed in this study. Whether G6PD-CLTC contributes to axonal regeneration through these pathways remains unknown. More precise spatial mapping of G6PD–CLTC complexes in neurons using super-resolution imaging techniques would help address this question (39). While our study focuses on DRG neurons, the conservation of G6PD–CLTC interaction across neuronal subtypes remains to be determined. Given the distinct regenerative capacities of peripheral *versus* CNS neurons, it would be highly valuable to investigate whether forced expression of G6PD in CNS neurons could overcome their intrinsic regenerative barriers. This approach holds significant translational potential for treating CNS injuries and neurodegenerative diseases.

## Conclusion

In summary, this work uncovers a previously unrecognized role for G6PD in axonal regeneration: beyond its canonical metabolic activity, G6PD serves as a direct regulator of CME through interaction with CLTC, thereby enabling the membrane dynamics essential for neuronal repair. Targeting the G6PD–CLTC axis may thus represent a promising therapeutic strategy to enhance nerve regeneration in a range of neuropathological conditions.

## Experimental procedures

### Animals

Sprague-Dawley rats (180–220 g) were obtained from the Experiment Animal Center of Nantong University. All the animals used in the experiments were maintained in a pathogen-free facility at 23 to 24 °C under a 12-h light, 12-h dark regimen with free access to food and water.

### Sciatic nerve crush injury

Following anesthesia by an intraperitoneal injection of 40 mg/kg sodium pentobarbital, the left hind limb of rats was shaved and disinfected. A small incision was made to expose the sciatic nerve, and the nerve was crushed at a site 10 mm above the bifurcation into the tibial and common fibular nerves with a pair of forceps for 10 s, repeated three times at the same location. The injury site was marked with a 9-0 nylon suture as previously described (40). After surgery, animals were placed in a warm environment with stable temperature and humidity to facilitate postoperative recovery.

### AAV constructs and packaging

The shRNA sequences targeting rat G6PD or CLTC were designed as follows: G6PD-shRNA1, CCTGGCCAAGAAGAAGATT; G6PD-shRNA2, CCTCATGGTGCTGAGATTT; and G6PD-shRNA3, GGGATGGAGTACCCTTCAT; CLTC-shRNA, CGTGTTCTTGTAACCTTTA. AAV2/8 vectors for either G6PD knockdown or overexpression were packaged by OBIO Technology. Viral titers were determined by RT-qPCR and were approximately 5 × 10^12^ genome copies per milliliters.

### Intrathecal injection

For intrathecal injection, adult rats were anesthetized, and the lumbar region was shaved to expose the injection site. A total volume of 10 μl of viral solution was slowly injected into the cerebrospinal fluid between the L5 and L6 vertebrae using a 10-μl Hamilton syringe. Following injection, the needle was kept in place for an additional 2 min to facilitate proper diffusion of the virus. Rats were then allowed to recover for 2 weeks to ensure sufficient viral expression prior to any behavioral assessments or surgical interventions.

### RNA extraction and quantitative real-time PCR

Total RNA from DRG tissues was extracted using TRIzol regent (Sigma-Aldrich, T9424), and cDNA was synthesized with the HiScript III first Strand cDNA Synthesis Kit (+ gDNA wiper) (Vazyme, R312–02) following the manufacturer’s instructions. Quantitative PCR was performed using SYBR Green Master Mix (Vazyme, Q141-02) on a Step One Plus Real-time PCR System (Applied Biosystems). GAPDH was used as the internal control, and gene expression levels were calculated using the 2^−ΔΔCT^ method. The primers used in this study are listed in Table S3.

### Primary DRG neuron culture and *in vitro* neurite regrowth model

DRGs from rats were dissected in ice-cold Hibernate-A medium (Gibco, A1247501) and digested with 0.5 mg/ml collagenase (Sigma-Aldrich, C0130) for 2 h, followed by treatment with 0.125% trypsin (Sigma-Aldrich, 4049) for 10 min at 37 °C. Cells were gently triturated with 1-ml pipette tips in Neurobasal medium (Gibco, 10888022) supplemented with 2% B27 (stemcell, 05711) and 1% glutamine (Gibco, A2916801), filtered through a 70-μm strainer (Biosharp, BS-70-XBS), and purified using 15% BSA (BioFroxx, 4240GR100) density gradient centrifugation. Finally, neurons were plated on poly-L-lysine–coated (Sigma-Aldrich, P4832) plates.

To mimic axonal injury *in vitro*, neurons at DIV3 were detached by gentle pipetting, resuspended, and replated onto poly-L-lysine–coated 24-well plates. After ∼16 h, neurons were fixed and subjected to immunofluorescence staining with Tuj1 to evaluate neurite regrowth.

### AAV infection of primary DRG neurons

Primary DRG neurons (∼10^4^ cells/well) were plated in 6-well plates. At 24 h, cultures were infected with 5 μl of AAV in antibiotic-free medium. After 12 to 16 h, the medium was replaced with fresh neurobasal medium containing B27, glutamine, and cytosine β-D-arabinofuranoside (Sigma-Aldrich, C1768) at a final concentration of 5 μM to inhibit non-neuronal proliferation. Seven days postinfection, neurons were replated onto 24-well plates. Cells were fixed 16 h later for immunofluorescence analysis.

### 6AN or Pitstop 2 treatment

Primary DRG neurons cultured for 3 days were detached and replated to mimic axonal injury. Different concentrations of 6AN (MedChemExpress, HY-W010342) or 20 μM Pitstop 2 (MedChemExpress, HY-115604) were added immediately after replating, and the neurons were maintained for indicated times prior to immunofluorescence staining.

### Immunohistochemical and immunocytochemical procedures

For immunohistochemistry, rats were perfused transcardially with PBS, followed by 4% paraformaldehyde (PFA). Dissected DRGs or sciatic nerve tissues were post-fixed in 4% PFA overnight at 4 °C, cryoprotected in 30% sucrose, embedded in OCT compound (Sakura, 4583), and sectioned at thickness of 20 μm using a cryostat. Tissue sections were permeabilized with 0.3% Triton X-100 and blocked as described above. Sections were then incubated overnight at 4 °C with primary antibodies: anti-Tuj1 (rabbit, 1:500, Abcam, ab18207), anti-Tuj1 (mouse, 1:500, R&D Systems, MAB1195), or anti-SCG10 (1:500, Abcam, ab210702), followed by Alexa Fluor–conjugated secondary antibodies (Invitrogen, A-11008, A-11001, A-11012, A-11005, A-21235) for 2 h at room temperature. Images were acquired under identical exposure settings to ensure consistency between experimental groups.

For immunocytochemistry, cultured DRG neurons were fixed with 4% PFA for 15 min, followed by permeabilization with 0.3% Triton X-100 (Sigma-Aldrich, T9284) in PBS for 10 min. After washing, cells were blocked in immunohistochemical blocking solution (Beyotime, P0102) for 1 h at room temperature. Neurons were then incubated overnight at 4 °C with the following primary antibodies to label specific proteins: anti-Tuj1 (rabbit, 1:1000, Abcam, ab18207), anti-Tuj1 (mouse, 1:1000, R&D Systems, MAB1195). The next day, cells were incubated with species-appropriate Alexa Fluor–conjugated secondary antibodies (Invitrogen, A-11008, A-11001, A-11012, A-11005, A-21235) for 2 h at room temperature. Nuclei were counterstained with DAPI.

The images were captured with a Zeiss Axio Imager M2 fluorescence microscope. To minimize variability between images, the intensity values of each cell or tissue were normalized to the background fluorescence signal, and mean values of intensities were calculated for each animal or sample using ImageJ (https://imagej.net/ij/). All measurements were performed blind to the experimental groups.

### Duolink PLA

Protein–protein direct interactions were assessed using the Duolink *in situ* PLA kit (Sigma-Aldrich, DUO92101) according to the manufacturer’s protocol. Cells were incubated with specific primary antibodies targeting the proteins of interest: anti-G6PD (1:200, Abcam, ab210702), and anti-CLTC (1:200, Cell Signaling Technology, 4796). After washing, cells were incubated with species-specific PLA secondary probes conjugated to oligonucleotides. When the two target proteins were in close proximity (<40 nm), the probes hybridized and were ligated, followed by rolling circle amplification. The resulting fluorescent puncta were visualized using a Zeiss LSM 900 confocal microscope.

### Co-IP assay

Co-IP of the protein samples was performed using the Pierce Classic Magnetic IP/Co-IP Kit (Thermo Fisher Scientific, 88804) according to manufacturer instructions. The lysates were incubated overnight at 4 °C with 1 μg of G6PD antibody (Abcam, ab210702) under gentle rotation. Protein A/G magnetic beads were then added to capture immune complexes. Following several washes, the bound proteins were eluted and subjected to MS analysis (Beyu Biotech) or Western blotting analysis. The 10% aliquot of the cell lysate was subjected to Western blotting analysis as an input control.

### MS analysis

Rat DRG tissues were subjected to label-free quantitative proteomic analysis. Two independent biological samples were analyzed, each measured in technical duplicate. Proteins were extracted in SDT lysis buffer (4% SDS, 100 mM DTT, 100 mM Tris–HCl, pH 7.6) and heated at 95 °C for 5 min to ensure complete denaturation and protease inactivation. Protein samples were processed using a filter-aided sample preparation protocol with 10 kDa molecular weight cut-off filters. After buffer exchange with 8 M urea in 150 mM Tris–HCl (pH 8.0), proteins were alkylated with 50 mM iodoacetamide for 30 min in the dark at room temperature. Samples were subsequently washed and digested overnight at 37 °C with sequencing-grade–modified trypsin (enzyme-to-protein ratio approximately 1:50). Peptides were collected by centrifugation, desalted using C18 StageTips, vacuum-dried, and reconstituted in 0.1% formic acid. Peptide concentration was determined by absorbance at 280 nm prior to LC–MS analysis. No specific enrichment for posttranslational modifications was performed. Peptides were separated using an Easy-nLC 1200 system (Thermo Fisher Scientific) coupled to a Q Exactive Plus mass spectrometer (Thermo Fisher Scientific). Peptides were first loaded onto a C18 trap column (100 μm × 20 mm, 5 μm, Dr Maisch GmbH) and then separated on an analytical C18 column (75 μm × 150 mm, 3 μm, Dr Maisch GmbH) at a flow rate of 300 nl/min. Mobile phase A consisted of 0.1% formic acid in water and mobile phase B consisted of 0.1% formic acid in 80% acetonitrile. Peptides were eluted with a 60-min gradient as follows: 2 to 5% B (0–2 min), 5 to 28% B (2–44 min), 28 to 40% B (44–51 min), 40 to 100% B (51–53 min), followed by 100% B (53–60 min). The mass spectrometer was operated in positive ion mode using data-dependent acquisition. Full MS scans were acquired in the Orbitrap over a mass range of 350 to 1800 *m/z* at a resolution of 60,000 (at *m/z* 200), with an automatic gain control target of 3 × 10ˆ6 and a maximum injection time of 50 ms. The top 20 most intense precursor ions were selected for higher-energy collisional dissociation with a normalized collision energy of 28 and an isolation window of 1.6 *m/z*. MS/MS spectra were acquired at a resolution of 15,000 with an automatic gain control target of 1 × 10ˆ5 and a maximum injection time of 50 ms. Dynamic exclusion was set to 30 s. Raw MS data were processed using MaxQuant (version 2.0.1.0, https://maxquant.org/). Spectra were searched against the UniProt Reference Proteome database for *Rattus norvegicus* (taxonomy ID 10116; 47,943 entries; downloaded March 29, 2023). A target-reverse database strategy was employed to estimate the false discovery rate. Trypsin was specified as the protease with up to two missed cleavages allowed. Carbamidomethylation of cysteine was set as a fixed modification, whereas oxidation of methionine and protein N-terminal acetylation were specified as variable modifications. The precursor mass tolerance was set to 20 ppm for the first search and 4.5 ppm for the main search, and the fragment ion mass tolerance was set to 20 ppm. The false discovery rate was controlled at 1% at the peptide-spectrum match, protein, and modification site levels. Proteins identified with at least one unique peptide were retained for downstream analysis. Label-free quantification was performed using the MaxQuant label-free quantification algorithm with default parameters.

### Western blotting analysis

Proteins were extracted from DRG tissues or primary cultured neurons using RIPA lysis buffer (Beyotime, P0013B) and quantified using a BCA Protein Assay Kit. Equal amounts of protein (30 μg) were separated by SDS-PAGE and transferred onto polyvinylidene fluoride membranes. Membranes were blocked in 5% nonfat milk in Tris-buffered saline with 0.1% Tween-20 for 1 h at room temperature, followed by overnight incubation at 4 °C with primary antibodies: anti-GAPDH (1:2500, Proteintech, 60004-1-Ig), anti-G6PD (1:1000, Proteintech, 66373-1-Ig), anti-G6PD (1:1000, Abcam, ab210702), or anti-CLTC (1:1000, Cell Signaling, 4796). After washing, membranes were incubated with horseradish peroxidase–conjugated secondary anti-rabbit (1:2000, Vazyme, RA1008-01) or anti-mouse (1:2000, Vazyme, RA1009-01) antibodies for 2 h at room temperature. Protein bands were detected using enhanced chemiluminescence reagents and imaged with a chemiluminescent detection system. The protein expression level was quantified densitometrically using ImageJ. The relative protein expression was calculated after normalization to GAPDH.

### NADP^+^/NADPH ratio measurement

NADP^+^/NADPH ratio was analyzed using NADP/NADPH Detection Kit (Sigma-Aldrich, MAK038) according to manufacturer instructions. Briefly, DRG tissues or cultured DRG neurons were lysed in 100 μl NADPH extraction buffer on ice for 10 min, followed by sonication and centrifugation at 1000×*g* for 10 min. For total NADP (NADP__total_), 50 μl supernatant was transferred to a 96-well plate. For NADPH alone, samples were heated at 60 °C for 30 min to decompose NADP^+^. Each well received 100 μl Master Reaction Mix, incubated 5 min at room temperature, followed by 10 μl NADPH Developer for 1 to 4 h. Absorbance was measured at 450 nm, and the reaction was terminated with 10 μl Stop Solution per well. All measurements were performed in triplicate and normalized to protein concentration.

$${\text{NADP}}^{+}{\text{/NADPH}}^{\text{ratio}}=\frac{{\text{NADP}}_{\mathrm{total}}-NADPH}{NADPH}$$

### Transferrin-endocytosis assay in primary DRG neurons

Transferrin endocytosis assay was performed as described previously in (41) with slight modifications. Prior to labeling, neurons were starved in neurobasal medium for 2 h, then incubated for 30 min at 37 °C in neuron culture medium containing 50 μg/ml human transferrin-Alexa Fluor 488 (Thermo Fisher Scientific, T13342). Cells were washed three times with ice-cold PBS, twice with ice-cold acid wash buffer (glycine-HCl, 150 mM NaCl, pH 2.2) to strip surface-bound transferrin, and again three times with ice-cold PBS. Neurons were then fixed with 4% PFA and processed for immunocytochemistry using rabbit anti-Tuj1 (1:1000, Abcam, ab18207) to label β-tubulin III; nuclei were counterstained with DAPI. Images were captured on a Zeiss LSM 900 confocal microscope, and fluorescence intensity was quantified with ImageJ.

### Functional enrichment analysis

Functional enrichment analysis of G6PD-specific binding proteins was performed using the R package clusterProfiler (version 4.12.6) under the KEGG pathway annotation (42). Multiple testing correction was conducted using the Benjamini–Hochberg method, and pathways with adjusted *p* < 0.05 were considered significant. The top enriched pathways were visualized with the ggplot2 package in R.

### Thermal sensitivity assessment

Thermal nociceptive responses were measured using the Hargreaves apparatus (Ugo Basile) as previously described (18). Rats were placed in transparent enclosures on a glass surface and allowed to acclimate for 15 min prior to testing. The radiant heat source was applied to the plantar surface of the hind paw, and the withdrawal latency was recorded. The device was set to 30% intensity, with a 30-s cut-off time to prevent tissue damage. Preoperative tests were performed to screen for any abnormal responses, defined as a consistent failure to withdraw the paw within 30 s across three trials. No such abnormalities were observed in the tested animals. Each paw was tested three times with an interval of at least 10 min between trials.

### Statistical analysis

All animals and neuronal cultures were randomly assigned to groups before experimental manipulation. Sample size was calculated with G∗Power 3.1 software, and values were set at *p* = 0.05, power = 0.8, and an effect size estimated from the previous experiments or pilot studies (43, 44, 45). Biological replicates are indicated in figure legends. Data acquisition and analysis were performed blind to group identity. GraphPad Prism 8 (GraphPad Software, https://www.graphpad.com/scientific-software/prism/) was used for analysis. Unless otherwise indicated, two-group comparisons were analyzed by unpaired Student’s *t*-tests, and multiple-group comparisons by one-way or two-way ANOVA, followed by Bonferroni or Dunnett *post hoc* tests. Data are shown as mean ± SD, and *p* < 0.05 was considered statistically significant.

## Data availability

Sequencing data of the rat DRGs at 0 h, 1 day, 4 days, and 7 days after sciatic nerve crush injury were stored in the NCBI database with the accession number PRJNA547681 (SRP200823). All other data generated during this study are included in the article and supporting files. Original data from this study can be obtained upon request from the corresponding author.

## Ethics statement

All experimental procedures involving animals were conducted in accordance with protocols approved by the Institutional Animal Care and Use Committees of Nantong University (approval ID: P20230224-014).

## Supporting information

This article contains supporting information.

## Conflict of interest

The authors declare that they have no conflicts of interest with the contents of this article.

## References

[1] Guijas C., Montenegro-Burke J.R., Warth B., Spilker M.E., Siuzdak G. (2018). Metabolomics activity screening for identifying metabolites that modulate phenotype. Nat. Biotechnol..

[2] Guo D., Meng Y., Zhao G., Wu Q., Lu Z. (2025). Moonlighting functions of glucose metabolic enzymes and metabolites in cancer. Nat. Rev. Cancer.

[3] Faubert B., Solmonson A., DeBerardinis R.J. (2020). Metabolic reprogramming and cancer progression. Science.

[4] Masin L., Bergmans S., Van Dyck A., Farrow K., De Groef L., Moons L. (2024). Local glycolysis supports injury-induced axonal regeneration. J. Cell Biol..

[5] Yang C., Wang X., Wang J., Wang X., Chen W., Lu N. (2020). Rewiring neuronal glycerolipid metabolism determines the extent of axon regeneration. Neuron.

[6] Li F., Sami A., Noristani H.N., Slattery K., Qiu J., Groves T. (2020). Glial metabolic rewiring promotes axon regeneration and functional recovery in the central nervous system. Cell Metab..

[7] Hilton B.J., Griffin J.M., Fawcett J.W., Bradke F. (2024). Neuronal maturation and axon regeneration: unfixing circuitry to enable repair. Nat. Rev. Neurosci..

[8] Wu S., Xu J., Dai Y., Yu B., Zhu J., Mao S. (2023). Insight into protein synthesis in axon regeneration. Exp. Neurol..

[9] Dalla Costa I., Buchanan C.N., Zdradzinski M.D., Sahoo P.K., Smith T.P., Thames E. (2021). The functional organization of axonal mRNA transport and translation. Nat. Rev. Neurosci..

[10] He Z., Jin Y. (2016). Intrinsic control of axon regeneration. Neuron.

[11] Park K.K., Liu K., Hu Y., Smith P.D., Wang C., Cai B. (2008). Promoting axon regeneration in the adult CNS by modulation of the PTEN/mTOR pathway. Science.

[12] Liu K., Lu Y., Lee J.K., Samara R., Willenberg R., Sears-Kraxberger I. (2010). PTEN deletion enhances the regenerative ability of adult corticospinal neurons. Nat. Neurosci..

[13] Brauer A.U., Savaskan N.E., Kuhn H., Prehn S., Ninnemann O., Nitsch R. (2003). A new phospholipid phosphatase, PRG-1, is involved in axon growth and regenerative sprouting. Nat. Neurosci..

[14] Wang D., Zheng T., Zhou S., Liu M., Liu Y., Gu X. (2023). Promoting axon regeneration by inhibiting RNA N6-methyladenosine demethylase ALKBH5. eLife.

[15] Jiang C., Lu Y., Zhu R., Zong Y., Huang Y., Wang D. (2023). Pyruvate dehydrogenase beta subunit (Pdhb) promotes peripheral axon regeneration by regulating energy supply and gene expression. Exp. Neurol..

[16] Gong L., Wu J., Zhou S., Wang Y., Qin J., Yu B. (2016). Global analysis of transcriptome in dorsal root ganglia following peripheral nerve injury in rats. Biochem. Biophys. Res. Commun..

[17] Li S., Xue C., Yuan Y., Zhang R., Wang Y., Wang Y. (2015). The transcriptional landscape of dorsal root ganglia after sciatic nerve transection. Sci. Rep..

[18] Mao S., Chen Y., Feng W., Zhou S., Jiang C., Zhang J. (2022). RSK1 promotes mammalian axon regeneration by inducing the synthesis of regeneration-related proteins. PLoS Biol..

[19] Saijilafu Hur E.M., Liu C.M., Jiao Z., Xu W.L., Zhou F.Q. (2013). PI3K-GSK3 signalling regulates mammalian axon regeneration by inducing the expression of Smad1. Nat. Commun..

[20] Shin J.E., Geisler S., DiAntonio A. (2014). Dynamic regulation of SCG10 in regenerating axons after injury. Exp. Neurol..

[21] Abe N., Borson S.H., Gambello M.J., Wang F., Cavalli V. (2010). Mammalian target of rapamycin (mTOR) activation increases axonal growth capacity of injured peripheral nerves. J. Biol. Chem..

[22] Weng Y.L., An R., Cassin J., Joseph J., Mi R., Wang C. (2017). An intrinsic epigenetic barrier for functional axon regeneration. Neuron.

[23] Aurora A.B., Khivansara V., Leach A., Gill J.G., Martin-Sandoval M., Yang C. (2022). Loss of glucose 6-phosphate dehydrogenase function increases oxidative stress and glutaminolysis in metastasizing melanoma cells. Proc. Natl. Acad. Sci. U. S. A..

[24] Hervera A., De Virgiliis F., Palmisano I., Zhou L., Tantardini E., Kong G. (2018). Reactive oxygen species regulate axonal regeneration through the release of exosomal NADPH oxidase 2 complexes into injured axons. Nat. Cell Biol..

[25] Jin X., Li X., Li L., Zhong B., Hong Y., Niu J. (2022). Glucose-6-phosphate dehydrogenase exerts antistress effects independently of its enzymatic activity. J. Biol. Chem..

[26] Kaksonen M., Roux A. (2018). Mechanisms of clathrin-mediated endocytosis. Nat. Rev. Mol. Cell Biol..

[27] Alam M.S. (2018). Proximity ligation assay (PLA). Curr. Protoc. Immunol..

[28] Fang J., Jiang W., Zhao W., Wang J., Cao B., Wang N. (2024). Endocytosis restricts dendrite branching via removing ectopically localized branching ligands. Nat. Commun..

[29] Shi G., Hao D., Zhang L., Qin J., Tian G., Ma B. (2021). Endocytosis-associated patterns in nerve regeneration after peripheral nerve injury. J. Orthop. Transl..

[30] von Kleist L., Stahlschmidt W., Bulut H., Gromova K., Puchkov D., Robertson M.J. (2011). Role of the clathrin terminal domain in regulating coated pit dynamics revealed by small molecule inhibition. Cell.

[31] Markelonis G.J., Oh T.H., Park L.P., Azari P., Max S.R. (1985). Receptor-mediated uptake of labeled transferrin by embryonic chicken dorsal root ganglion neurons in culture. Int. J. Dev. Neurosci..

[32] Mote R.D., Yadav J., Singh S.B., Tiwari M., V S.L., Patil S. (2020). Pluripotency of embryonic stem cells lacking clathrin-mediated endocytosis cannot be rescued by restoring cellular stiffness. J. Biol. Chem..

[33] Liu R., Zhou B. (2025). Harmine promotes axon regeneration through enhancing glucose metabolism. J. Biol. Chem..

[34] Cappellini M.D., Fiorelli G. (2008). Glucose-6-phosphate dehydrogenase deficiency. Lancet.

[35] Meng Q., Zhang Y., Hao S., Sun H., Liu B., Zhou H. (2022). Recent findings in the regulation of G6PD and its role in diseases. Front. Pharmacol..

[36] Camblor-Perujo S., Kononenko N.L. (2022). Brain-specific functions of the endocytic machinery. FEBS J..

[37] Eichel K. (2025). Endocytosis in the axon initial segment: roles in neuronal polarity and plasticity. Curr. Opin. Neurobiol..

[38] Hausott B., Forste A., Zach F., Mangger S., Haugsten E.M., Klimaschewski L. (2019). Endocytosis and transport of growth factor receptors in peripheral axon regeneration: novel lessons from neurons expressing lysine-deficient FGF receptor type 1 in vitro. Anat. Rec. (Hoboken).

[39] Tyagi S., Higerd-Rusli G.P., Akin E.J., Baker C.A., Liu S., Dib-Hajj F.B. (2024). Real-time imaging of axonal membrane protein life cycles. Nat. Protoc..

[40] Pan H.C., Cheng F.C., Chen C.J., Lai S.Z., Lee C.W., Yang D.Y. (2007). Post-injury regeneration in rat sciatic nerve facilitated by neurotrophic factors secreted by amniotic fluid mesenchymal stem cells. J. Clin. Neurosci..

[41] Kononenko N.L., Classen G.A., Kuijpers M., Puchkov D., Maritzen T., Tempes A. (2017). Retrograde transport of TrkB-containing autophagosomes via the adaptor AP-2 mediates neuronal complexity and prevents neurodegeneration. Nat. Commun..

[42] Xu S., Hu E., Cai Y., Xie Z., Luo X., Zhan L. (2024). Using clusterProfiler to characterize multiomics data. Nat. Protoc..

[43] Faul F., Erdfelder E., Buchner A., Lang A.G. (2009). Statistical power analyses using G∗Power 3.1: tests for correlation and regression analyses. Behav. Res. Methods.

[44] Au N.P.B., Wu T., Chen X., Gao F., Li Y.T.Y., Tam W.Y. (2023). Genome-wide study reveals novel roles for formin-2 in axon regeneration as a microtubule dynamics regulator and therapeutic target for nerve repair. Neuron.

[45] Decourt C., Schaeffer J., Blot B., Paccard A., Excoffier B., Pende M. (2023). The RSK2-RPS6 axis promotes axonal regeneration in the peripheral and central nervous systems. PLoS Biol..

