# Supplementary material for: G6PD facilitates axon regeneration via clathrin-mediated endocytosis
Source: J Biol Chem. 2026 Mar 4;302(4):111345. doi: 10.1016/j.jbc.2026.111345 (PMC13059121; doi:10.1016/j.jbc.2026.111345)
Supplement: Supplemental Figures [file mmc1.docx]

**SUPPORTING INFORMATION**

**G6PD facilitates axon regeneration via clathrin-mediated endocytosis**

Chunyi Jiang^1^, Xinyi Liu^1^, Hui Li^1^, Yan Lu^1^, Qianqian Cao^1^, Bin Yu^1, *^, Susu Mao^1, *^

^1^Jiangsu Key Laboratory of Tissue Engineering and Neuroregeneration, Key Laboratory of Neuroregeneration of Ministry of Education, Affiliated Hospital of Nantong University, Co-Innovation Center of Neuroregeneration, Nantong University, Nantong 226001, China

*Corresponding author:

Bin Yu: yubin@ntu.edu.cn

Susu Mao: maosusu@ntu.edu.cn


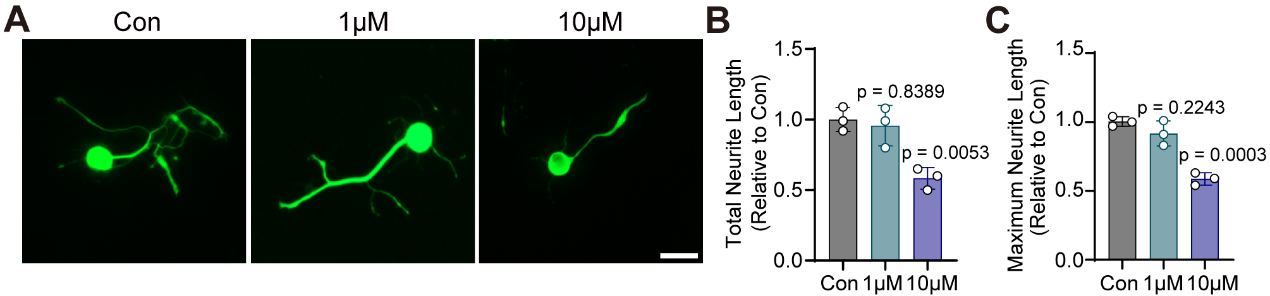


**Figure S1. Effect of the G6PD metabolic enzyme inhibitor 6-aminonicotinamide on axon regeneration of DRG neurons.** (A) Representative images of replated primary DRG neurons treated with 1 μM or 10 μM 6-aminonicotinamide (6AN), labeled with Tuj-1. DMSO treatment served as the control (Con). Scale bar, 20 μm. (B, C) Quantitative analysis of total and maximum neurite length per neuron in panel (A), normalized to Con group (mean ± S.D.; one-way ANOVA, Dunnett post hoc test, n = 3 biologically independent experiments).

**Table S1. Mass Spectrometry Dataset of Proteins Interacting with G6PD Protein.**

**Table S2. KEGG pathway enrichment analysis of genes specifically binding to G6PD.**

**Table S3. List of the primers used in qPCR.**
