# Supplementary material for: G6PD facilitates axon regeneration via clathrin-mediated endocytosis
Source: J Biol Chem. 2026 Mar 4;302(4):111345. doi: 10.1016/j.jbc.2026.111345 (PMC13059121; doi:10.1016/j.jbc.2026.111345)
Supplement: Table S3 [file mmc4.docx]

**Table S3. List of the primers used in qPCR.**

| **Gene** | **Forward primers** | **Reverse primers** |
| --- | --- | --- |
| G6pd | TGAGGACCAGATCTACCGCA | TCAAAATAGCCCCCACGACC |
| Pgls | GTCCTCACCATCGATCCCG | CAATGGGAGCCACGATCTTCT |
| Pgd | GGCCATCGCTGCAAAAGTAG | TCAAGTGGTAAGCCTCGCAG |
| Pfkm | TCTTCGGCCTGTAGAGGTTT | GTTTTAGCTCTCCTCCGCCG |
| Glut3 | CGAAGGTGACCCCATCTCTG | CCGCTCTTCCAACGTGTAGT |
| Hk2 | ACGGAGCTCAACCAAAACCA | CTTCCGGAACCGCCTAGAAA |
| Gapdh | ATGCCATCACTGCCACTCA | CCTGCTTCACCACCTTCTTG |
